# Cultural differences in joint attention and engagement in mutual gaze with a robot face

**DOI:** 10.1038/s41598-023-38704-7

**Published:** 2023-07-19

**Authors:** Serena Marchesi, Abdulaziz Abubshait, Kyveli Kompatsiari, Yan Wu, Agnieszka Wykowska

**Affiliations:** 1 Social Cognition in Human-Robot Interaction, Italian Institute of Technology, Genova, Italy; 2 Robotics and Autonomous Systems Department, A*STAR Institute for Infocomm Research, Singapore, Singapore

**Keywords:** Psychology, Engineering

## Abstract

Joint attention is a pivotal mechanism underlying human ability to interact with one another. The fundamental nature of joint attention in the context of social cognition has led researchers to develop tasks that address this mechanism and operationalize it in a laboratory setting, in the form of a gaze cueing paradigm. In the present study, we addressed the question of whether engaging in joint attention with a robot face is culture-specific. We adapted a classical gaze-cueing paradigm such that a robot avatar cued participants’ gaze subsequent to either engaging participants in eye contact or not. Our critical question of interest was whether the gaze cueing effect (GCE) is stable across different cultures, especially if cognitive resources to exert top-down control are reduced. To achieve the latter, we introduced a mathematical stress task orthogonally to the gaze cueing protocol. Results showed larger GCE in the Singapore sample, relative to the Italian sample, independent of gaze type (eye contact vs. no eye contact) or amount of experienced stress, which translates to available cognitive resources. Moreover, since after each block, participants rated how engaged they felt with the robot avatar during the task, we observed that Italian participants rated as more engaging the avatar during the eye contact blocks, relative to no eye contact while Singaporean participants did not show any difference in engagement relative to the gaze. We discuss the results in terms of cultural differences in robot-induced joint attention, and engagement in eye contact, as well as the dissociation between implicit and explicit measures related to processing of gaze.

## Introduction

Humans are involved in social interactions on a daily basis. The success of these interactions is based on a myriad of socio-cognitive mechanisms. One of those mechanisms is joint attention. Joint attention consists in following others’ directional signals (gaze, pointing) and attending jointly towards the same location or event in the environment^1^. Joint attention allows us to navigate all social interactions and, eventually, to attune with others^2,3^. Moreover, joint attention develops in the early stages of life, and it is necessary for other higher-order socio-cognitive mechanisms, such as understanding other’s mental states (e.g., thoughts, feelings)^4^. In classic laboratory experiments, joint attention has been studied by means of the gaze-cueing paradigm. Typically, in such paradigm, a face (or a face-like stimulus) is presented on a screen. Initially, the stimulus is presented with a gaze directed straight toward the observer. Subsequently, the eyes gaze to a given position on the screen (often, towards the left or right with respect to the face). This is defined as the directional “gaze cue” ^5–8^. After a certain period of time, defined as stimulus-onset asynchrony (SOA), a target appears either at the same (validly cued) or different than gazed-at location (invalidly-cued). Classic results in this task show faster Reaction Times (RTs) for the detection or discrimination of validly cued targets, compared to invalidly cued targets. The difference in RTs between invalidly and validly cued targets represent the gaze-cueing effect (GCE) and is related to attentional orienting in response to the directional cue. The GCE can be elicited by various types of eye-like stimuli^9,10^. For example, Takahashi and colleagues used 2D images of pareidolia faces, showing that these stimuli can elicit the gaze cueing as well. Along the same line, Admoni and colleagues used 2D images of toys^11^ and Quadflieg and colleagues used 2D images of dolls^9^. Both show that these stimuli can evoke the GCE. Recently, GCE has been shown to be elicited also by artificial agents^11–16^, morphed faces, avatars ^17^ and robots^18–20^. For example,Wykowska and colleagues^16^ reported that high order beliefs, such as the belief that eyes of a robotic face are controlled by a human, can impact behavioral gaze cueing effects and the Event Related Potentials (ERPs) related to joint attention. Similarly, Caruana and colleagues^17^ investigated the influence of attributing mental states to an avatar on the consequent gaze cueing paradigm. The authors report that the attribution of mental states influences the way social cues produced by the avatar are processed at the neural level. Finally, Abubshait and colleagues investigated the effect of perceptual ambiguous faces (morphs between human and robot faces) could interfere with the top-down control in a counterpredictive gaze cueing task. The authors found that categorically ambiguous faces affect the top-down control on the distribution of the cognitive resources, as they create cognitive conflict. In sum, there is a strong corpus of literature using non-human stimuli (including robot faces) as central cuer in attention orienting.

Following this corpus of literature, the present work aimed to investigate how joint attention elicited by a robot face is culture-dependent, and whether potential modulations of GCE related to cultural embedding would be observed when top-down control mechanisms are loaded with an orthogonal task (and thus made less available for the joint attention task).

### Why robot faces?

Social robotics is a fast-growing field aiming at designing robots for application in healthcare, elderly care, and education^21–23^. Social robots are meant to assist humans in daily tasks and provide companionship and/or support in training activities. However, before one can employ robots in such social contexts, one needs to understand what are the key ingredients of robots’ behavioral design and physical appearance that are necessary for inducing social attunement and good social communication with human users. Social attunement can be understood as (often nonverbal) tuning of basic mechanisms that our brain has developed in—and for interactions with others. Joint attention is one of such mechanisms. It allows two (or more) interaction partners to attend—in a time-aligned manner—to the same location or event in the shared environment. If one points to a location in space and the other attends to that location, they both understand that they are referring to the same object in the discussion. As such, joint attention allows for sharing the grounds for joint communication and for social attunement. Joint attention is usually relatively automatically evoked by other humans. However, it is not yet clear what factors influence the establishment of joint attention with robots. As argued earlier, robots might evoke joint attention in some circumstances, but in some others, this might not be the case.

As social robots are meant to populate our environments in the not-so-far future, one needs to examine all possible factors that are at play in establishing social attunement.

### Why joint attention in the context of cognitive control?

GCE has been observed in contexts where the gaze is not a reliable signal to predict the subsequent location of the target, and thus showing its reflexive component ^5,24^. Nonetheless, several authors showed that GCE is prone to a top-down modulation. The GCE depends on a social context and relevance^12,16,25,26^, familiarity of the gazer^25^, reliability of the cue^27,28^, social status^29^, and ingroup/outgroup status of the gazer^30^. Also the perception of being in competition with the gazer affects the GCE^31^ .Finally, and importantly for the purposes of the present study, Kompatsiari et al.^18,20^ showed that engaging participants in eye contact or not before displaying the directional cue affects the GCE. In more detail, the authors found that in the absence of a need for top-down strategic control (non-predictive cues, 50% cue validity) the social signal of eye contact enhanced the default GCE by prolonging the attentional prioritization of the gazed-at location. The modulation was observed when the measurement was taken after a sufficient amount of time (1000 ms SOA), while no modulation occurred when the measurement was taken earlier in time (500 ms SOA). Instead, both gaze conditions elicited a GCE. Taken together, there is ample evidence that GCE is a combination of a bottom-up (i.e., automatic/reflexive) and a top-down (i.e., contextual/social) components^12,32–34^.

Given the interplay of bottom and top-down components in the GCE, researchers have tried to disentangle the factors that can elicit the factors, allowing for one component to take precedence over the other. The amount of resources dedicated to the attentional systems, depends on the cognitive control mechanisms, usually studied introducing task irrelevant facets^35,36^. Indeed, when cognitive control mechanisms are overloaded, the results show slower Reaction Times (RTs) when the task-relevant and irrelevant information are incongruent, as for the GCE. To further understand whether adding cognitive load during a joint attention task would affect the priority given to the bottom-up/top-down components, several authors used the GC paradigm. For example, Pecchinenda and Petrucci^35^ investigated how cognitive load (induced by asking participants to complete a counting task at the beginning of each block) would influence the GCE where the central cuer would express different emotions (i.e., angry, happy or neutral), with non-predictive cues. The authors report that they found a modulation of the GCE for the angry and happy faces, while no GCE was found for the neutral face. The authors argue that, the shortage of cognitive resources due to an orthogonal task, leads to a different prioritization of the top-down (i.e., social salience of the facial expression) and bottom-up (i.e., directional cues) components. Recently, Abubshait and colleagues^12^ investigated whether categorically ambiguous faces could evoke cognitive load (Experiment 1). Subsequently whether the cognitive load elicited by these ambiguous faces could affect the top-down components in a counterpredictive GC task (Experiment 2) and, finally, whether exposing participants to the ambiguous faces before the task would alleviate the cognitive control mechanism and thus freeing cognitive resources. The authors report that categorically ambiguous stimuli can indeed evoke cognitive conflict, that affects the top-down control on attentional orienting in a counterpredicitive gaze-cueing paradigm. Importantly, pre-exposure to the stimuli prior to the gaze-cueing task can attenuate the loss of cognitive resources.

In sum, the amount of cognitive resources available when joint attention mechanisms are active, will impact the interplay between top-down and bottom-up components and the priority with which the information are processed^32^. In the context of joint attention with robots, it is interesting to understand whether robot faces evoke GCE even when top-down control is depleted due to load induced by an orthogonal task. If GCE effects are observed under a high cognitive load condition, it would mean that orienting attention in response to robot face is relatively automatic.

### Why culture?

In addition to the discussed factors that could modulate joint attention, there is another layer of factors that can influence humans’ mechanisms of social cognition: cultural differences. Humans are embedded in cultures and thus, cultural differences affect the way we perceive and process social signals^37–41^. Recent literature showed that social attention, and in particular gaze cues, are modulated by participants' cultural background^42,43^. Furthermore, the amount and duration of eye contact might differ across cultures both in everyday life and in experimental settings^38,44^.

In cross-cultural studies on joint attention, East Asian individuals have been shown to engage less in mutual gaze compared to Western Caucasians during, for example, business relationships^45^ (Japanese vs. Americans) or doctor/patient conversation^46^ (Chinese vs. Canadians). Furthermore, on an explicit level, British individuals and Italians have rated eye contact in a conversation as more important compared to individuals from Japan or Hong Kong. The effect of reduced mutual gaze in East Asians has been attributed to the fact that averted gaze symbolizes showing respect in East Asian cultures, pointing to a kind of “gaze avoidance” sociocultural norm^47,48^. On the other hand, more recently Haensel et al.^49,50^, showed that both cultural groups (British/Irish vs Japanese participants) engaged more their attention in the conversational partner’s face during listening compared to speaking and during an introductory task compared to a storytelling game. However, in contrast to classical literature reporting that East Asian individuals usually engage less in mutual gaze when compared to Western Caucasian individuals, Haensel et al.^50^ observed that contextual factors can challenge this pattern. Eastern Asian individuals have been shown to engage in mutual gaze more than Western Caucasian individuals in specific settings (such as in storytelling task)^50^. Looking at a face longer when listening and answering questions (compared to speaking or storytelling) can be attributed to an attempt to use gaze aversion to reduce the cognitive load present in the more cognitively-demanding tasks (i.e., speaking and storytelling)^51^. Indeed direct and mutual gaze can be more cognitive demanding, leading to the development of strategies to manage the cognitive load since a very young age (i.e., 5 years old)^52–54^, from both neuro-typically developed children and neuro-divergent children^52,53^.

Since cultural differences affect processing gaze in general, one should expect that culture affects also how we process social signals from artificial agents^55^. Despite the number of studies that investigated cross-cultural differences in human–robot interaction^39,56,57^, the role of culture in joint attention effects has not been clarified. To address this question in the present study, we first needed to select an Eastern population that would be the representative of the Eastern culture. Our reasoning to choose Singapore as representative of the South-Asian sample, was driven by our willingness to adhere to inclusion criteria: most of the cross-cultural investigations in cultural psychology (and human–robot interaction) are addressing the population of bigger nations such as China or Japan (at least in the field of HRI^56^). This can lead to a selection bias(as historically was done with the Western population), leaving South-Asia out of the scientific investigation radar.

## Aim of study

As mentioned above, the aim of our study was to investigate GCE induced by a robot face across different cultural groups and under two different social gaze conditions, namely eye contact vs. averted gaze with the robot face before the directional gaze. The latter factor has been shown to modulate GCE^19^ and might also be culture-dependent. In addition, we manipulated cognitive load/stress in order to examine the contribution of bottom-up/top-down mechanisms to the effects of interest.

Regarding cultural differences, we did not have a directional hypothesis regarding the basic GCE. We expected that GCE could be comparable across cultures, but we reasoned that it might also be the case that there are cultural differences in robot-induced GCE, as robots might be more likely perceived as social entities in some cultures and less in others.

Regarding the eye contact manipulation, we hypothesized that eye contact would enhance GCE in the sample drawn from Italian culture, where eye contact has been associated as a positive element of social interactions^58^. This hypothesis was based also on previous literature^18,20,59^. Kompatsiari and colleagues found that eye contact with a robot elicited GCE while averted gaze did not. In Kompatsiari et al.^20^, after having conducted a series of experiments where both validity and SOA were manipulated, this effect was explained with reference to the idea that in the 1000 ms SOA condition with averted gaze, the transient bottom-up-driven attentional orienting effect washes away with time. In the case of mutual gaze, this effect is prolonged, due to the impact of a strong social signal (the mutual gaze). Since the present study involved also 1000 SOA and 50% validity, we expected that also here, we would observe GCE in the mutual gaze condition and no (or attenuated) GCE in the averted gaze.

In the sample of Singaporean individuals, we hypothesized that the effects would be either similar to the Italian sample (under the assumption that cultural differences do not play a role in artificial agents’ gaze processing), or we should observe a different pattern, if cultural differences play a role in modulatory effects of social signals on attentional orienting.

Finally, regarding the stress/cognitive load manipulation, our reasoning followed Kompatsiari et al.^18^.where the authors argued that the social factor affecting GCE is only potent enough to modulate (prolong in time) the top-down component of GCE. Thus, we expected that the differential GCE as a function of mutual/averted gaze would be observed more prominently in a low stress condition (where the top-down component was at play) and less clearly in a high stress condition, where more automatic processes were expected (due to limited cognitive control mechanisms being loaded with the high stress orthogonal task^12^). The high stress condition was expected to elicit similar effects to the default bottom-up GCE as observed in Kompatsiari et al.^20^ with 500 ms SOA and nonpredictive cues.

We did not have directional hypotheses related to cultural differences impacting the stress/cognitive load manipulation and its impact on GCE.

Lastly, based on Kompatsiari et al.^18,20^ procedure, we tested our participants’ engagement during the task. In the Italian sample, we expect to replicate the results reported by Kompatsiari and colleagues^18,20^, showing a higher engagement rating for the eye contact blocks compared to the no eye contact blocks. In the Singaporean sample, given the lower tendency to engage in eye contact^49^, we expected no difference in the social engagement ratings between the two gaze conditions.

## Materials and methods

### Participants

Participants in both Nations were recruited via an email advertisement to mailing lists. Inclusion criteria were matched across the two Countries as follows: (a) age range: 21–45 (the lower boundary was set on the consent legal age in Singapore, the upper boundary was set following the ethical protocols approved in Singapore); (b) no previous history of epilepsy or neurological disorders; c. being right-handed.

The sample size was matched with previous studies investigating the GCE effect with the iCub robot as a gazer^15,20^ or in cross-cultural contexts^42,60^. Based on these criteria, *w*e collected data from N = 30 Singaporean participants (F = 15, mean age = 32.77, SD = 9.26) and N = 32 Italian participants (F = 19, mean age = 26.66, SD = 5.88). Data from one Singaporean participant were excluded due missing data in the stress questionnaire in S2. Two additional participants were excluded due to a low accuracy score (< 80% of correctly identified letters). The final Singaporean sample was N = 27 (F = 14, mean age = 32.70, SD = 9.37). Two Italian participants were excluded due to familiarity with the stress-inducing math tasks and 3 participants were excluded due to a low accuracy score (< 80% of correctly identified letters). The final Italian sample was N = 27 (F = 16, mean age = 26.15, SD = 5.24). The study was approved by both Singaporean and Italian local Ethical Committees (A*STAR’s IRB and Comitato Etico Regione Liguria, respectively) and was conducted in accordance with the Code of Ethics of the World Medical Association (Declaration of Helsinki). Each participant provided written informed consent before taking part in the experiment. All participants were naïve to the purpose of this experiment but were debriefed after the experiment. All participants received compensation for their participation: a number of coupons equal to 30SGD for each participant in the Singaporean sample and 15 Euros for each participant of the Italian sample.

### Stimuli and apparatus

**Figure 1:**
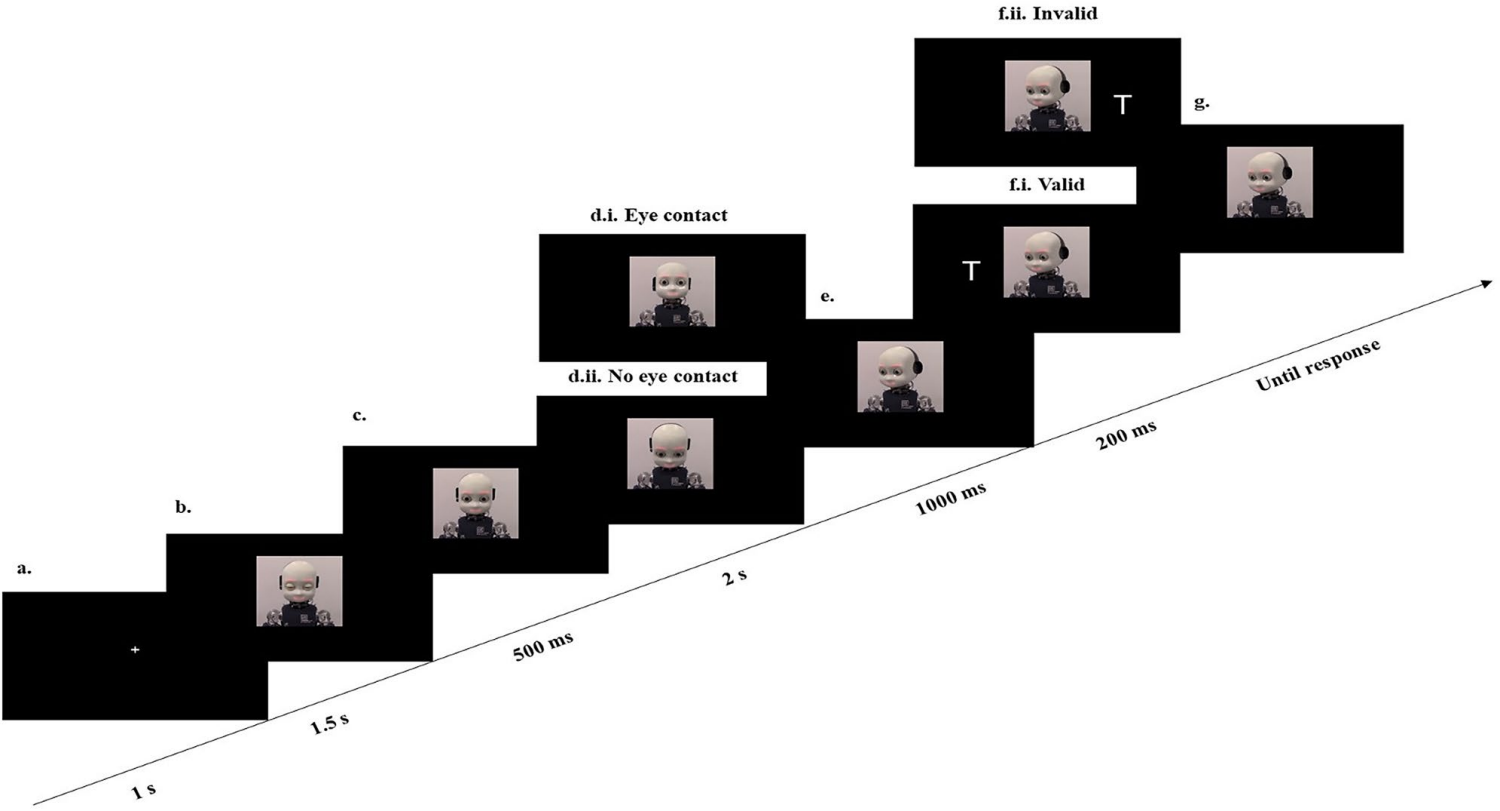
Trial sequence. (**a**) A fixation cross appears for 1 s. (**b**) iCub’s face with eyes closed is presented for 1.5 s. (**c**) iCub’s face with eyes open is presented in the same position. (**d**) After 500 ms, a face with direct (eye contact: d.i.) or downward gaze (eye contact c.ii) appears for 2 s. (**e**) Then, iCub looks laterally towards a potential target location. (**f**) after 1000 ms the target letter (Τ or V) appears for 200 ms. The robot’s gaze is non-predictive of the target location (50% validity, Valid condition: f.i., Invalid condition: f.ii.). (**g**) The participant (not shown) discriminates against the target by pressing T or V.

Pictures were frames taken from videos of iCub performing a similar task in its embodied version^19^. iCub’s gaze was directed to five different positions: eye contact–towards participant eyes, no eye contact–looking downwards, left–towards the target letter on the left side of the screen, right–towards the target letter on the right side of the screen, and resting– between eye contact and no eye contact gaze condition. The design was drawn from a similar online experiment (see^20^ Experiment 1 in Supplementary material (SM1) for a similar procedure), and coded in Psychopy 2021.2.3^61^ where units for visual stimuli were set to ‘height’ to account for possible diversity of the screens between the two labs across Italy and Singapore. The size of iCub’s image was set to 0.95 × 0.6 and appeared at the center of the screen. The target stimuli consisted of two letters appearing on the screen either on the left or on the right of the central iCub picture (a “V” or a “T” letter with height: 0.21 height units). To ensure the same perceived motion in both conditions, in the “eye contact” condition, the robot was moving from resting position (neck pitch: − 12 degrees) to straight (neck pitch: 0 degrees) while in the “no eye contact” condition, the robot was moving from resting position (neck pitch: − 12 degrees) to downward neck pitch: − 24 degrees (see Fig. 1). The experiment was run on the same DELL Latitude laptop (Intel Core i5) that was connected to a 27-inch monitor, where the stimuli were appearing. A standard keyboard was connected to the laptop to allow participants’ response. The same protocol was followed in the labs in Italy and in Singapore. The room in Italy and the room in Singapore were both dimly lit. All materials and testing conditions were identical between the two laboratories.

### Design and procedure

Each trial of the gaze-cueing task started with a fixation cross for 1 s to ensure that participants attended the center of the screen where the robot’s face would be presented. Then, the picture of iCub was presented with eyes closed at resting position. After 1.5 s, the picture of iCub with eyes open at the resting position appeared for 500 ms. Then, the picture with the straight gaze (eye contact condition) or downward gaze (no eye contact condition) was presented for 2 s. Next, the picture of iCub’s shifted gaze to the screen’s left or right side was presented for 1000 ms. A target letter appeared on one of the sides (Y position: − 0.5; X position: left/right = − 0.02/0.02 height units) of the screens for 200 ms. The letters disappeared, but the picture with iCub’s shifted gaze remained until response. The cue-target validity was non-predictive (50% validity), meaning that the robot would gaze at the same location where the letter would appear in 50% of the trials (valid trials) and in the other 50%, iCub would gaze at the opposite direction (invalid trials). Participants were instructed to stay fixated on the robot’s face (even if it would move its eyes). They were asked to identify the letter that would appear on one of the robot’s sides by pressing the corresponding letter on a standard keyboard with their middle fingers of each hand. The position of the left/right finger with respect to the V and T keys was counterbalanced. If participants did not press any key within 1500 ms after the target onset, the trial expired, and participants’ response was categorized as incorrect. eye contact/no eye contact was manipulated across blocks. The block order was pseudo-randomized, and it was counterbalanced across participants. Half of the participants started the experiment with an “eye contact” block, while the other half with a “no eye contact” block. In total, the experiment consisted of 16 blocks of 16 trials each.

In addition to the GCE, we also assessed the subjective experience of the two types of gazesby asking participants at the end of each block: “How much did you feel engaged with the robot”. Participants responded to this question by rating their engagement on a 10-point Likert scale. At the end of every block, participants were reminded to fixate at the robot’s face, and respond to the target identification task as fast and as accurately as possible.

Finally, in addition to our main manipulation of interest, we introduced a stress manipulation, to see whether cultural differences affect more the bottom-up or the top-down components of GCE. However, this manipulation was only subsidiary to the main aim of the study.

Before starting the gaze cueing experiment, participants were asked to answer 7 questions on a 10-point Likert scale assessing their stress level (S1). After this questionnaire, participants performed the Math Effort Test (MET^62^). Once the MET was completed, participants’ stress level was assessed again (S2) with the same questions as those presented in S1. Immediately thereafter, participants performed the gaze cueing task. Once half of the experiment was completed (8 blocks), participants completed the MET for the third time (S3). Finally, participants performed the second half of the gaze cueing task.

As it is not possible to guarantee that “no stress” condition would induce no stress at all (performing an experiment in the lab might be stressful enough to some individuals), we did not have a “no stress” condition in our design, but rather addressed the level of stress by splitting our samples according to their reported subjective level of stress. In other words, our “control” group was not a no-stress manipulation condition, but rather subjectively reported “low-stress” group, with the same stress manipulation as the experimental “high-stress” group. More specifically, participants were split according to their reported stress level score. This procedure was done first averaging S1 and S2 and, secondly, by splitting participants over the mean in the “High Stress” group and participants below the mean in the “Low Stress” group. Finally, we calculated the GCE for each participant by subtracting participant's RTs in the valid trials from participant's RTs in the invalid trials for both the mutual gaze condition and the avoiding gaze condition. Sphericity corrections were applied when necessary. All data were preprocessed and analyzed with R (4.2.0) and JASP (v. 0.16.2).

## Preprocessing and analyses pipeline

We preprocessed our data first by excluding speed outlier trials (< 50 ms and > 800 ms) to ensure that any variability due to the different mean age of the groups was detected. After this procedure, trials that were above 2 standard deviations with respect to the individual overall mean averaged across all conditions were considered outliers and, thus, excluded (11.60% of the total trials, final N of trials = 12,221). Then, we conducted a Mixed Factor Analysis of Variance (ANOVA), where participants’ RTs were averaged across conditions and considered as dependent variable and a 2 (Nationality) × 2 (Gaze type) × 2 (Validity) design was adopted.

To examine the impact of cultural embedding on the way type of gaze is subjectively experienced, we analyzed the subjective engagement reports using the Wilcoxon Signed Rank Test.

## Results

### Gaze Cueing results

**Figure 2:**
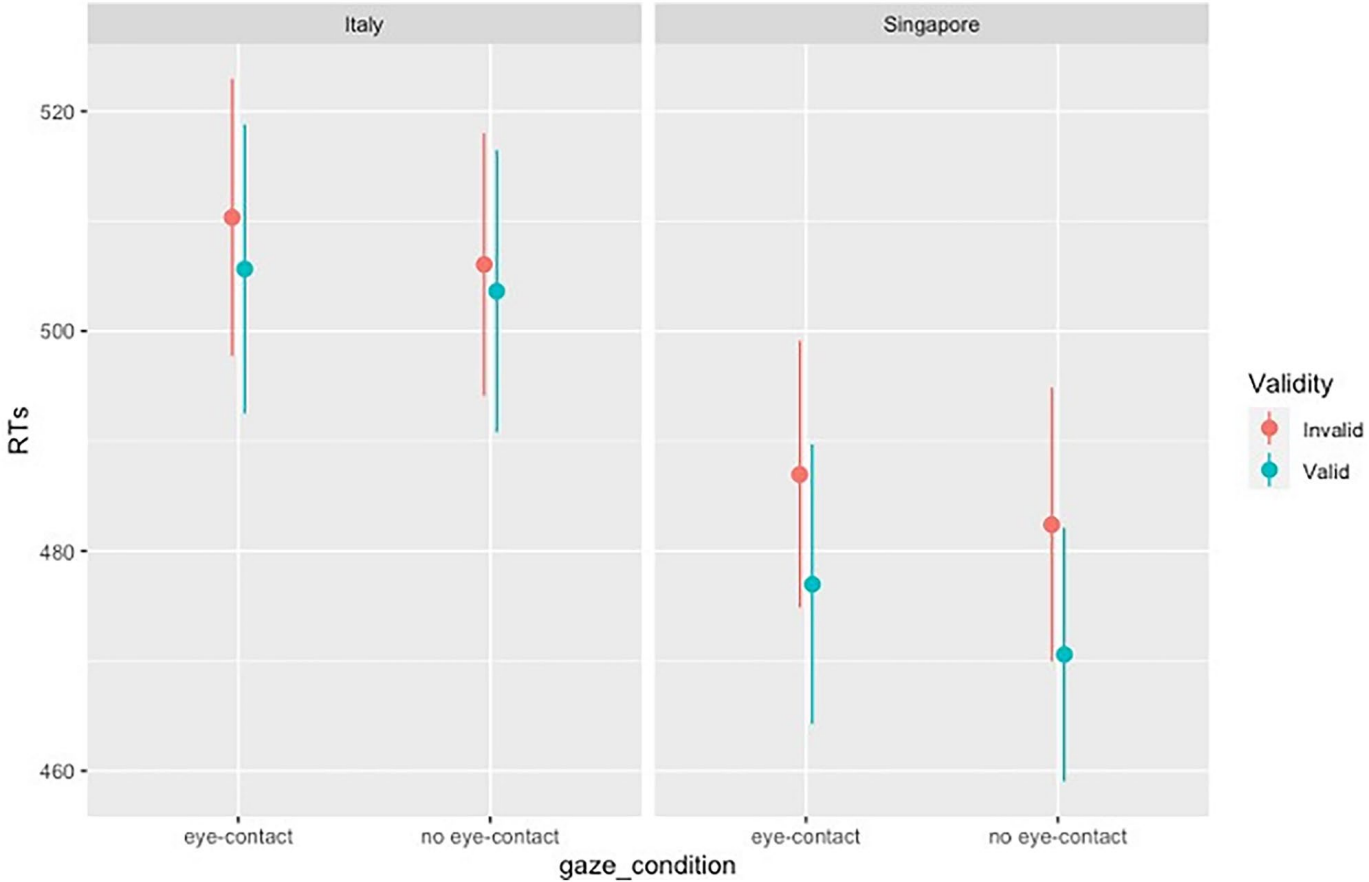
RTs as a function of Validity (valid vs. invalid), Gaze type (eye contact vs. no eye contact) and nationality (Italy vs. Singapore). Error Bars indicate Standard Errors of the mean.

Once data were preprocessed, we run a Mixed-factors Analysis of Variance (ANOVA) to investigate how participants’ nationality and gaze type (eye contact vs. no eye contact) influence GCE. Gaze type (eye contact vs no eye contact) and Validity (valid vs invalid) were considered as a within-subjects factors with two levels each, while Nationality (Singapore vs Italy) as between-subjects factor with two levels. Mean Reaction Times and standard deviations per each condition are reported in Table 1. The main effect of *Gaze type* emerged as significant [F(1,52) = 5.96, *p* = 0.018, η_p_^2^ = 0.1], showing that participants were in general faster in no eye contact , relative to eye contact gaze [M_no-eye contact_ = 490.67, SD_no-eye contact_ = 64.25; M_eye contactl_ = 494.98_,_ SD_eye contacl_ = 66.21]. In addition, the main effect of *Validity* was significant [F(1,52): 18.05, *p* =  < 0.001, η_p_^2^ = 0.26], showing that participants were faster for valid as compared to invalid trials [M_invalid_ = 496.44, SD_invalid_ = 64.04; M_valid_ = 489.21_,_ SD_valid_ = 66.29]. Additionally, the interaction between Validity and Nationality emerged as significant [F(1,52): 4.65, *p* = 0.036, η_p_^2^ = 0.08] (Fig. 1). Post-hoc comparisons showed that this interaction was driven by Singaporean participants being significantly faster in the valid, relative to the invalid, trials [t = − 4.53, p_holm_ =  < 0.001, SE = 2.41]. This effect was not present in the Italian sample [t = − 1.48, p_holm_ = 0.43, SE = 2.41] (Fig. 2). The three-way interaction of Gaze type, Validity and Nationality was not significant. As the statistical power might have been insufficient to detect a three-way interaction effect, we conducted an additional Bayesian analysis which confirmed the lack of the three-way interaction effect, see Supplementary Materials. All comparisons are reported in the Supplementary Materials.

**Table 1:** Mean RTs per condition.

Gaze type	Nationality	Validity	Mean	SD
Eye contact	Italy	Valid	505.65	68.33
		Invalid	510.33	65.50
	Singapore	Valid	476.98	66.05
		Invalid	486.98	62.91
No eye contact	Italy	Valid	503.62	66.67
		Invalid	506.05	62.08
	Singapore	Valid	470.58	59.98
		Invalid	482.40	64.67

### Engagement ratings

**Figure 3:**
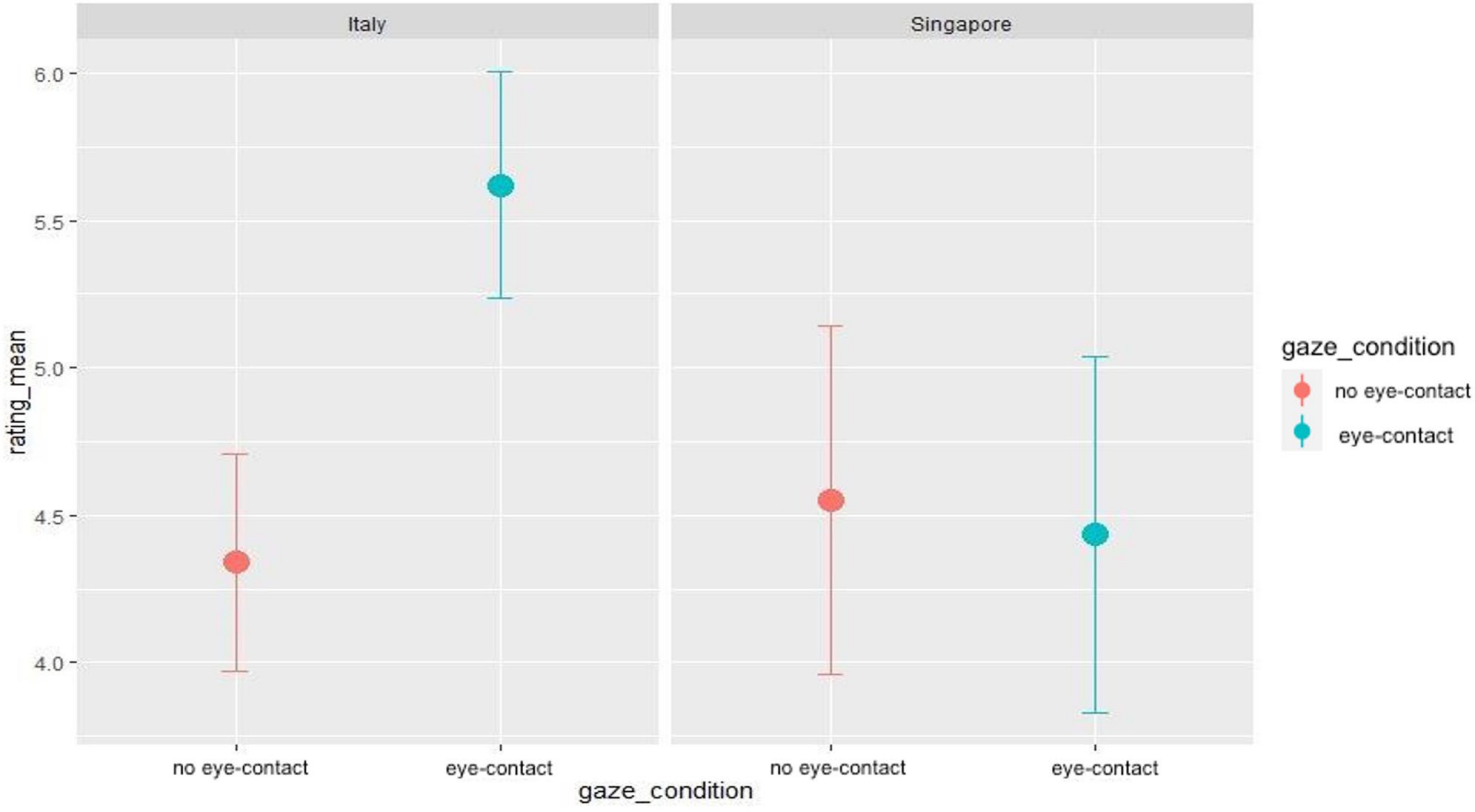
Rating scores from Italian and Singaporean participants. Error Bars indicate Standard Errors of the mean.

Italian participants rated the eye contact gaze as more engaging compared to the no eye contact condition [*V* = 323, *p* =  < 0.001 (*M*_eye contactl_ = 5.62, SD = 1.95, *M*_no-eye contact_ = 4.34, SD = 1.88), while Singaporean participants did not show any statistical difference in their rating scores [*V* = 54, p = 0.18 (*M*_eye contactyl_ = 4.43, SD = 2.96, *M*_no-eye contact_ = 4.55, SD = 2.90) (see Fig. 3).

## The effect of cognitive load/stress manipulation on the GCE

### Reported Stress Level

First, we checked that participants’ reported stress levels were significantly different across the three time points. To do so, we run a Mixed-factors Analysis of Variance (ANOVA) considering the Stress_Time as repeated measure factor and Nationality as between-subjects factor with two levels (Italy vs. Singapore). As data violated the sphericity assumption [Maulchly’s W = 0.82, *p* = 0.007, Greenhouse–Geisser ε = 0.85], we applied the Greenhouse–Geisser correction. The main effect of Stress_Time emerged as significant [F(1.70,52): 10.71, *p* =  < 0.001, η_p_^2^ = 0.12]. The interaction between Stress_Time and Nationality was not significant [F(1.7,52): 0.05, *p* = 0.933, η_p_^2^ =  < 0.001]. Post-hoc comparisons using Holm correction revealed that S1 differed from both S2 [t = − 4.30, p_holm_ =  < 0.001, SE = 0.29] and S3 [t = − 3.63., p_holm_ =  < 0.001, SE = 0.29]. No significant difference emerged between S2 and S3 [t = 0.67, p_holm_ = 0.447, SE = 0.29] (see Table 2).

**Table 2:** Reported stress scores.

Nationality	Stress_time	Mean	SD
Italy	S1	2.38	1.44
	S2	3.60	1.25
	S3	3.35	1.59
Singapore	S1	2.03	1.12
	S2	3.28	1.58
	S3	3.14	1.85

### GCE and individual stress level

**Figure 4:**
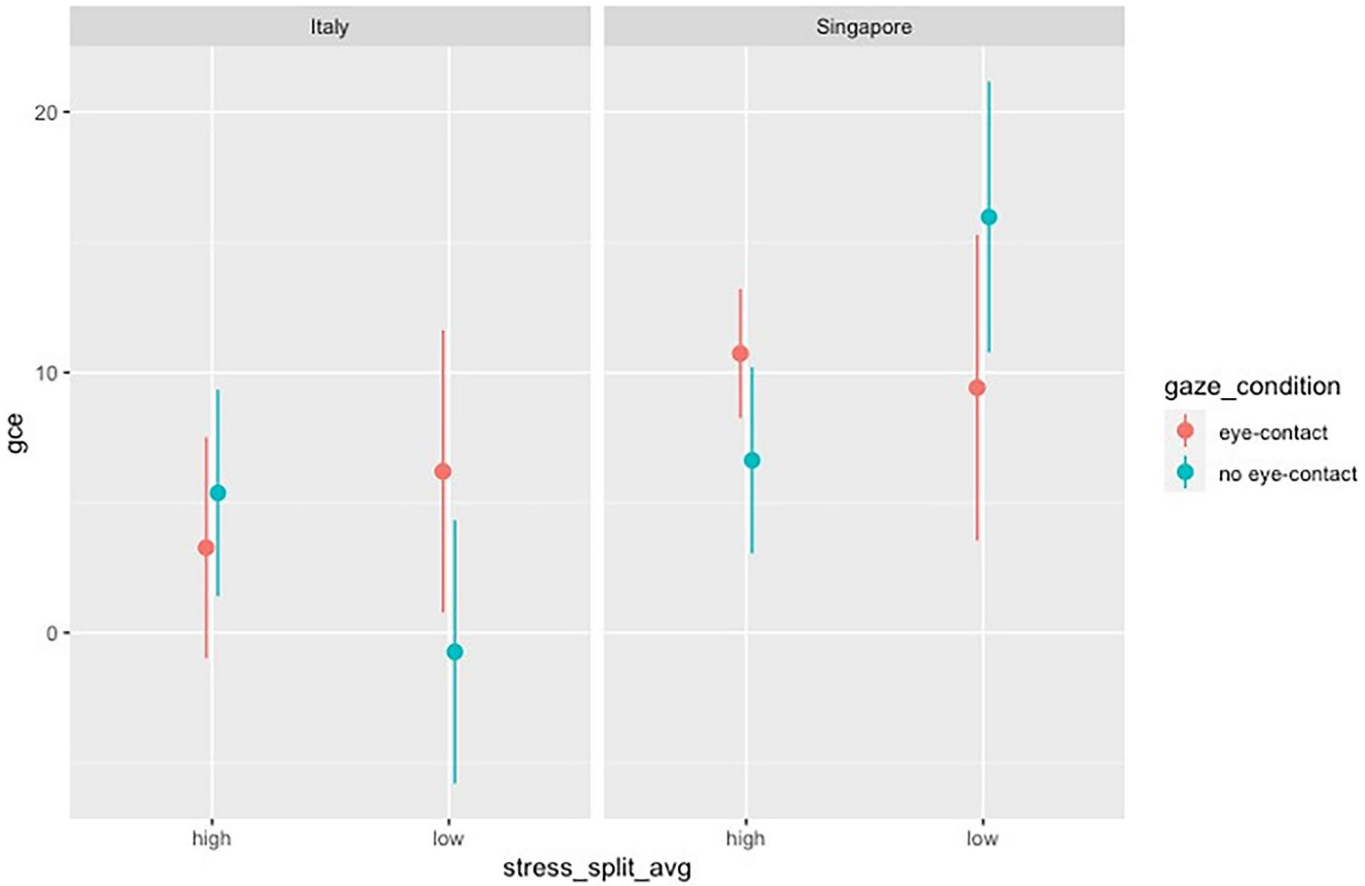
GCE as a function of Nationality (Italy vs. Singapore) and split according to the reported stress level (High vs. Low). Error Bars indicate Standard Errors of the mean.

Once data were preprocessed, we run a Mixed Analysis of Variance (ANOVA) with Satterthwaite’s method of degrees of freedom estimation to investigate how participants stress level and nationality influence GCE. *Gaze type* (mutual vs. no avoiding) and *Stress level* (high vs. low) were considered as a within-subjects factors with two levels each, while *Nationality* (Singapore vs. Italy) as between-subjects factor with two levels. The main effect of Nationality emerged as significant [F(1,50): 4.55, *p* = 0.03, η_p_^2^ = 0.04]. Post-hoc comparisons confirmed that the Italian sample had a lower GCE in general when compared to the Singaporean sample (t = − 2.13, *p*_tukey_ = 0.03, SE = 3.35, see Table 3 for a summary of the mean GCE in each condition). No other effects or interactions reached the level of statistical significance (See Fig. 4).

**Table 3:** Descriptives—gce.

Gaze_type	Nationality	Stress level	Mean	SD	N
No-eye contact	Italy	High	5.372	14.836	14
		Low	− 0.729	18.238	13
	Singapore	High	6.627	12.396	12
		Low	15.970	20.171	15
Eye-contact	Italy	High	3.274	15.878	14
		Low	6.201	19.542	13
	Singapore	High	10.728	8.585	12
		Low	9.412	22.712	15

## General discussion

The present study aimed at testing whether robot-induced GCE is culture-dependent, modulated by social gaze (eye contact vs. averted gaze) and sufficiently automatic to be observed also when cognitive control is loaded with an orthogonal (stressful) task. In addition, to explore how different types of gaze affect subjective experience of the gaze, depending on the cultural embedding, we analysed the subjective reports of engagement as a function of gaze type (eye contact/no eye contact) and culture (Singapore/Italy).

Our results confirmed previous literature that showed faster RTs for valid trials relative to invalid trials. Moreover, our participants showed a general tendency to show longer RTs for the eye-contact condition, which shows that eye contact held their attention, thereby resulting in a difficulty disengaging from the direct gaze (for similar results in humans see^63–65^. Most importantly for the purposes of our study, the main effect of validity was modulated by nationality and was observed only in the Singaporean sample. This confirms a general difference in processing directional gaze cues, which is exhibited by a robot avatar, where Singaporean participants showed a higher tendency to engage in joint attention with an artificial agent, relative to Italian participants. This might suggest that East Asian cultures are more prone to treating the robot faces as social, and thereby are more likely than European samples to employ towards artificial agents socio-cognitive mechanisms that have been developed for interactions with conspecifics (other humans). This is, however, only a speculation and should be examined further in future research. Of note is that previous literature^18–20^ showed that GCE in response to robot’s gaze can be observed also in Italian samples. The lack of validity effect in the Italian sample of the present study might be due to the fact that the robot face was presented only as a 2D stimulus on the screen, thereby reduced in ecological validity and social salience. Similarly, eye contact/no eye contact gaze did not modulate the GCE in either sample of participants. This might also be due to the artificial and non-social nature of the robot face presented on the screen. Reports in literature show that an embodied version of the same robot elicits differential effects of gaze type on GCE^18–20^. Finally, one needs to consider the choice of a long SOA adopted in our design, as this is a crucial parameter for interpreting gaze-cueing effects^66^. While long SOAs could attenuate (or even eliminate) cueing effects, or turn them into an inhibition of return effect ^67^, this specific choice in our design, this specific choice in our design was motivated by the observation of a series of studies of Kompatsiari and colleagues^18,20^ where the modulation of GCE by eye-contact with a robot was found only in an SOA of 1000 ms. This was interpreted as an effect of a prolonged prioritization of the validly cued location through the social signal of eye-contact. In the context of those previous results, we decided to use also 1000 ms SOA in our design.

Interestingly, subjective engagement ratings showed that Italian individuals rated higher the eye contact condition, compared to the no eye contact condition, confirming our hypothesis. This was not observed for the Singaporean sample. Higher engagement ratings for eye contact, relative to no eye contact, have been observed in previously, also in Italian samples^18,20^. However, it is interesting that the effect is not observed for the Singapore sample. This shows that although Singaporean participants showed a larger GCE than Italian participants, they did not express larger engagement with the eye contact of the robot, at the explicit level, in line with our expectations. On the other hand, unexpectedly the Italian sample was more sensitive to the eye contact of the robot at the explicit level, but did not show GCE. The lack of difference in the engagement ratings as a function of type of gaze in the Singaporean sample paralleled the GCE to some extent, as the Singaporean sample of participants showed validity effects of equal size for both gaze type conditions. Thus, it seems that the Singaporean sample was not sensitive to the gaze type manipulation, which might be due to a lower tendency to engage in eye contact for the Asian cultures^49^. On the contrary, the Italian sample was sensitive to the type of gaze at the explicit level but this was not paralleled by a modulatory effect of gaze type on GCE. This suggests that engagement and experience of gaze type at the explicit level relies on a different mechanism than the gaze cueing effects.

Finally, we conducted an exploratory analysis by splitting the samples based on their reported stress level, and the results confirmed only the impact of cultural embedding on the GCE. However, no interaction with the cognitive load/level of stress has been observed. As this is an incidental finding resulting from an exploratory analysis, we could only speculate that this suggests that the GCEs observed in the Singaporean sample reply substantially on bottom-up effects, as they occurred even in the condition when cognitive control mechanisms were loaded with an orthogonal task. Of note is an alternative explanation that perhaps the impact of the additional orthogonal task was not long-lasting enough to show effects in the gaze cueing task performed after the stress manipulation.

## Limitations

Not all our results were in line with our hypotheses. First, eye contact did not modulate gaze-cueing effects in any of the samples. We based our hypotheses on embodied interactions and we expected that screen-based stimuli would induce similar effects. However, this is not necessarily true, since embodied eye contact with another agent might elicit different behavioral, neural, and physiological responses compared with a screen-based direct gaze^68–71^. Therefore, no eye contact modulation might have arisen from the fact that the gaze was presented on the screen. Second, against our hypothesis, the Italian sample did not show a GCE overall. This is a striking result since gaze-cueing effects have been consistently elicited with robot’s faces. As mentioned earlier, this result might also arise from the fact that the gazer was a robot face and it was presented as a 2D stimulus. This suggests that robot faces do not automatically induce faze following in more Western cultures. However, these unexpected results should be further investigated in future studiesRelated to engagement ratings, we replicated our results in the Italian sample, i.e., participants found more engaging the eye contact condition compared to no eye contact. The Singaporean participants, however, rated the two gaze conditions equally. This is also a result that requires further research to understand the dissociation between the GCE and subjective engagement ratings. Regarding the effect of cognitive load/stress manipulation, Finally, it remains to be further examined whether the fact that we did not observe any impact of stress/cognitive load on the effects of interest is due to the automatic nature of the observed effects or rather inefficiency of the manipulation to impact the orthogonal gaze cueing task.

## Conclusions

In this study, our primary goal was to investigate how cultural embedding (Western Europeans vs East Asian) affects attentional orienting elicited by robot’s directional gaze and following a social gaze (eye contact or not). Furthermore, we aimed to investigate how explicit judgments related to engagement differ across cultures for robot’s eye contact or not. Finally, we introduced stress manipulation in order to examine whether and to what extent robot-induced gaze following is an automatic process. Our results showed that Singaporean participants are more likely to engage in joint attention with a robot face presented on the screen, while for Italian participants, this kind of stimulus might not be sufficient to induce gaze following. This might be related to the degree with which a simplified (2D) robot avatar is perceived as a social stimulus, which seems to be culture dependent. Such result has implications for the design of social robots, as it suggests that cultural factors need to be taken into account for understanding how “social” a simple robot behavior appears, which, in consequence, impacts social attunement^72^.

## Supplementary Information

Supplementary Information.

## Data Availability

Data are available at the following public OSF repository: https://osf.io/mrz57/.
