# Supplementary material for: Cultural differences in joint attention and engagement in mutual gaze with a robot face
Source: Sci Rep. 2023 Jul 19;13:11689. doi: 10.1038/s41598-023-38704-7 (PMC10356937; doi:10.1038/s41598-023-38704-7)
Supplement: Supplementary file 1 — Supplementary Information. [file 41598_2023_38704_MOESM1_ESM.docx]

**Supplementary Materials** **Cultural differences in joint attention and engagement in mutual gaze with a robot face**

Serena Marchesi^1,2^, Abdulaziz Abubshait^1^, Kyveli Kompatsiari^1^, Yan Wu^2^ and Agnieszka Wykowska^1*^

^1^ Social Cognition in Human-Robot Interaction, Italian Institute of Technology, Genova, Italy

^2^ Robotics and Autonomous Systems Department, A*STAR Institute for Infocomm Research, Singapore

*Corresponding Author

First, in order to ensure that the RTs from the two samples were comparable and were not affected by differences in the means age, we run an independent sample t-test (normality parameters of the data were met). Results confirmed no differences in the mean RTs between the two samples: [t(52)= 1.57, p= 0.12, SE=17.33, Cohen’s d= 0.43]. Thus, we can conclude that differences emerged from the other analysis are modulations of our manipulations.

| **SM Table 1. Group Descriptives** | | | | | | | | | | | |
| --- | --- | --- | --- | --- | --- | --- | --- | --- | --- | --- | --- |
|  | | Group | | N | | Mean | | SD | | SE | |
| Avg RTs |  | Italy |  | 27 |  | 506.44 |  | 64.77 |  | 12.47 |  |
|  |  | Singapore |  | 27 |  | 479.24 |  | 62.53 |  | 12.03 |  |
|  | | | | | | | | | | | |

**Bayesian Analysis**

To check whether the inferential analysis failed to show a three-way interaction due to a lack of power, we run Bayesian analyses on the main model. Results confirm that there is strong evidence against the three-way interaction (BFexcl = 25.4).

| **SM Table 2. Analysis of Effects** | | | | | | | | | | | |
| --- | --- | --- | --- | --- | --- | --- | --- | --- | --- | --- | --- |
| **Effects** | | **P(incl)** | | **P(excl)** | | **P(incl\|data)** | | **P(excl\|data)** | | **BF_excl_** | |
| Gaze Type |  | 0.737 |  | 0.263 |  | 0.760 |  | 0.240 |  | 0.884 |  |
| Validity |  | 0.737 |  | 0.263 |  | 0.995 |  | 0.005 |  | 0.015 |  |
| Gaze Type ✻  Validity |  | 0.316 |  | 0.684 |  | 0.108 |  | 0.892 |  | 3.815 |  |
| Nation |  | 0.737 |  | 0.263 |  | 0.599 |  | 0.401 |  | 1.878 |  |
| Gaze Type ✻  Nation |  | 0.316 |  | 0.684 |  | 0.138 |  | 0.862 |  | 2.886 |  |
| Validity ✻  Nation |  | 0.316 |  | 0.684 |  | 0.306 |  | 0.694 |  | 1.046 |  |
| Gaze Type ✻  Validity ✻  Nation |  | 0.053 |  | 0.947 |  | 0.002 |  | 0.998 |  | 25.401 |  |
|  | | | | | | | | | | | |
